# Minimal deviation mucinous adenocarcinoma of the uterine cervix that proved difficult to differentiate from endometrial cancer: A case report

**DOI:** 10.3892/ol.2014.2532

**Published:** 2014-09-12

**Authors:** YUKO NISHII, TAKESHI FUKUDA, KENJI IMAI, MAKOTO YAMAUCHI, YASUNORI HASHIGUCHI, TOMOYUKI ICHIMURA, TOMOYO YASUI, TOSHIYUKI SUMI

**Affiliations:** Department of Obstetrics and Gynecology, Osaka City University Graduate School of Medicine, Osaka 545-8585, Japan

**Keywords:** endometrial cancer, minimal deviation adenocarcinoma, adenoma malignum

## Abstract

Minimal deviation adenocarcinoma (MDA), also known as adenoma malignum of the uterine cervix, accounts for only ~1% of uterine cervical adenocarcinomas. Adenoma malignum of the uterine cervix was initially described by Gusserow in 1870. Using magnetic resonance imaging (MRI), MDA appears as multilocular lesions with solid components that extend from the endocervical glands to the deep cervical stroma. Cytological evaluation and biopsies have low detection rates, therefore, it is difficult to diagnose MDA accurately prior to treatment. The current study describes a rare case of MDA that was difficult to differentiate from endometrial adenocarcinoma of the corpus uteri preoperatively, as the endometrial biopsy results suggested a well-differentiated endometrioid adenocarcinoma and MRI did not show typical images for MDA. A total abdominal hysterectomy with bilateral salpingo-oophorectomy was performed under the diagnosis of endometrial cancer, and the mass was subsequently diagnosed as MDA of the uterine cervix by pathological examination of the hysterectomy specimen. Postoperatively, although two types of adjuvant chemotherapy were performed, the remaining tumor continued to grow, causing obstruction of the bilateral ureters and leading to bilateral hydronephrosis. The patient is currently alive with the disease 10 months following the surgery.

## Introduction

Minimal deviation adenocarcinoma (MDA), also known as adenoma malignum of the uterine cervix, accounts for only ~1% of adenocarcinomas of the uterine cervix. The main clinical manifestations are vaginal profuse, watery or mucoid discharge and irregular bleeding (1). In total, ~10% of MDA is accompanied by Peutz-Jeghers syndrome (2). Using magnetic resonance imaging (MRI), MDA appears as multilocular lesions with solid components that extend from the endocervical glands to the deep cervical stroma. Histopathologically, MDA is composed of mucinous, well-differentiated glands, deeply invading the cervical stroma, often surrounded by a desmoplastic reaction. Although MDA exhibits a benign histological appearance, it is typically characterized by aggressive clinical behavior. Cytological evaluation and biopsies have low detection rates, which delays the accurate diagnosis and leads to a poor prognosis.

The current study describes a rare case of MDA that was difficult to differentiate from endometrial adenocarcinoma of the corpus uteri preoperatively, as the endometrial biopsy results suggested well-differentiated endometrioid adenocarcinoma and MRI did not show typical images. Surgery was performed under the diagnosis of endometrial cancer, and the tumor was diagnosed as MDA of the uterine cervix following pathological examination of the hysterectomy specimen. Written informed consent was obtained from the patient.

## Case report

A 65-year-old female (gravida 2, para 0) visited a local clinic due to abnormal vaginal bleeding. A previous history of surgery for acute appendicitis was recorded at 20 years of age, and the patient’s family history was unremarkable. Mild ascites and swelling of an ovary were observed during transvaginal ultrasonography; therefore, the patient was referred to the Department of Obstetrics and Gynecology, Osaka City University Graduate School of Medicine (Osaka, Japan).

**Figure 1:**
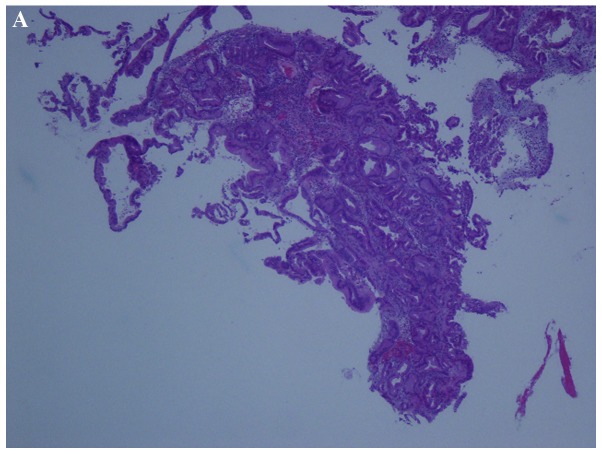

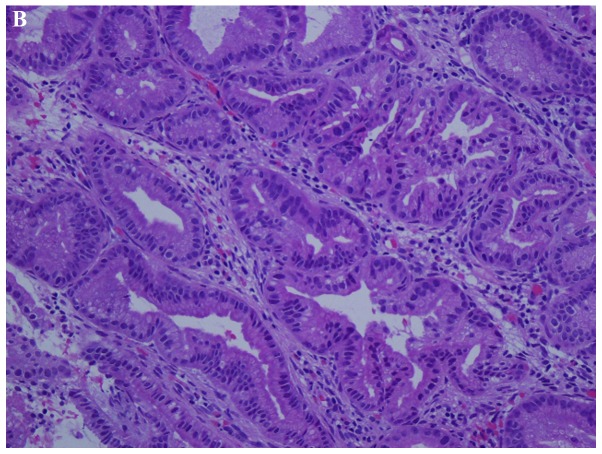
(A) Low-power and (B) high-power view of minimal deviation adenocarcinoma histopathology. The glands are well-differentiated and show minimal cytological atypia. The density of glands is increased and a number of glands are back to back [magnification, (A) ×20 and (B) ×200.]

**Figure 2:**
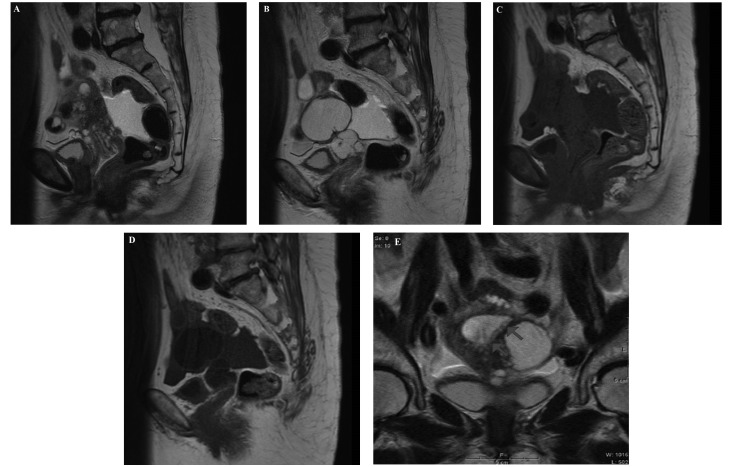
(A) T2-weighted sagittal magnetic resonance imaging revealing (A) no evident enlargement of the uterine cervix and (B) several high intensity cystic lesions in the uterine corpus and uterine cervix. (C) T1-weighted sagittal magnetic resonance image corresponding to Fig 2A. (D) T1-weighted sagittal magnetic resonance image corresponding to Fig. 2B. (E) T2-weighted coronal magnetic resonance image showing thickening of the endometrium (arrow).

On presentation, the patient exhibited a small volume of bloody vaginal discharge. The uterine corpus was enlarged to 85 mm in size and the uterine cervix was not enlarged or abnormal. Transvaginal ultrasonography revealed a small amount of ascites and mildly thickened endometrium. The fallopian tubes and ovaries exhibited no abnormalities. A cervical smear was negative for intraepithelial lesions or malignancy and an endometrial biopsy revealed a well-differentiated suspected endometrioid adenocarcinoma (Fig. 1). magnetic resonance imaging (MRI) revealed mucus retention in the endometrial cavity and multiple cysts from the uterine corpus to the uterine cervix, with low intensity on T1-weighted images and high intensity on T2-weighted images. No enlargement of the uterine cervix was observed. T2-weighted images revealed a high-intensity area in the endometrium of the uterine corpus (Fig. 2). A chest CT revealed the enlargement of the supraclavicular lymph nodes. Serum carbohydrate antigen (CA)-125 levels were 153 U/ml (normal, <35 U/ml) and CA19-9 levels were 44 U/ml (normal, <37 U/ml), while carcinoembryonic antigen and sialyl-Tn antigen levels were within the normal ranges.

**Figure 3:**
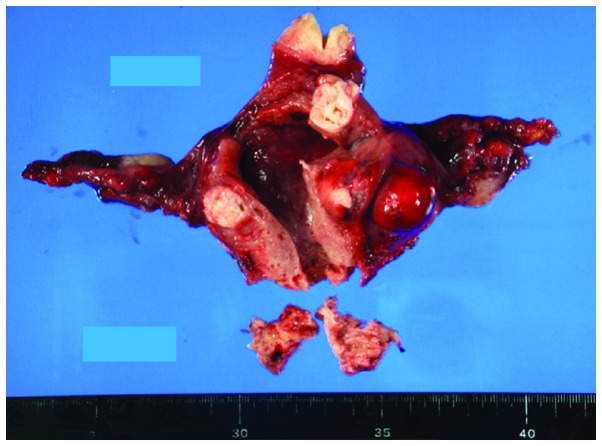
Extracted uterus and adnexa. The uterine cervix was extremely fragile and was damaged during hysterectomy.

**Figure 4:**
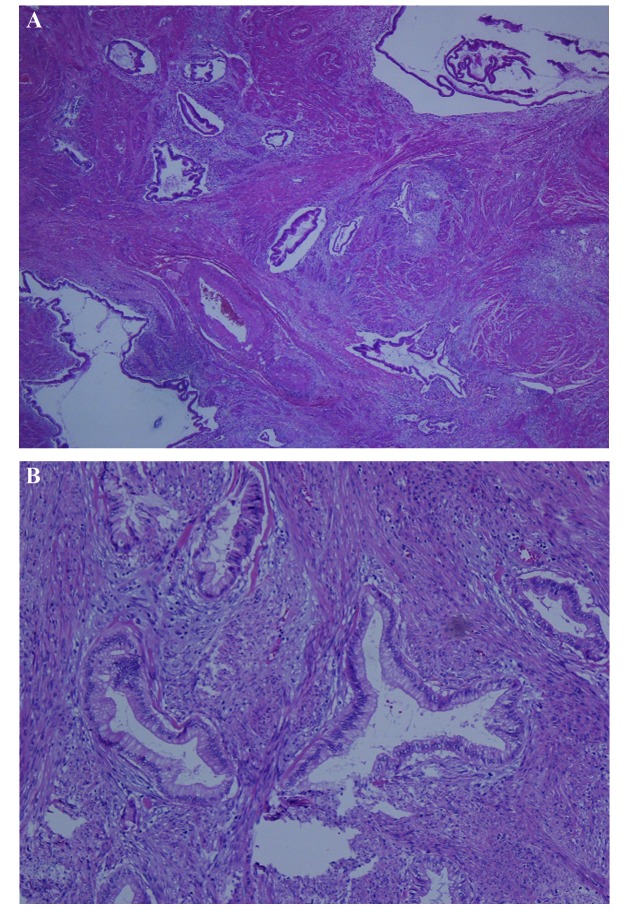
(A) Low-power and (B) high-power view of the multicystic lesions that resemble normal endocervical glands. The majority of glands had an irregular shape, cellular atypia and structural dysplasia. Branching-shaped endocervical glands infiltrated deep into the muscle [magnification, (A) ×100 and (B) ×200.

Based on the diagnosis of advanced endometrial adenocarcinoma of the corpus uteri, an abdominal total hysterectomy was performed with bilateral salpingo-oophorectomy and left supraclavicular lymph node biopsy. Intra-abdominal dissemination was observed and intraoperative peritoneal cytology was positive. The uterine cervix was extremely fragile and was damaged during the hysterectomy; therefore, the affected tissue was resected as much as possible. However, further tumors remained. Macroscopically, the uterine corpus was enlarged to goose-egg size and the tumor was located in the uterine cervix and uterine corpus. The tumor was predominantly present in the uterine cervix. The endometrial surface was moderately irregular (Fig. 3) and microscopically, deeply infiltrating mucinous adenocarcinoma, composed of well-formed glands arranged in an irregular fashion, was identified. The nuclear and architectural abnormalities were generally minimal; however, limited areas exhibited a desmoplastic stromal reaction, indicating a malignant nature (Fig. 4). A final diagnosis of MDA of the uterine cervix was determined. Extension of tumors to the uterine corpus, fallopian tubes and ovaries was identified and the left supraclavicular lymph node was positive for metastasis. The tumor was designated as pT4NXM1, according to the Union for International Cancer Control TNM classification (7th edition) (3).

In total, six courses of chemotherapy containing irinotecan and cisplatin were administered postoperatively; however, the growth of the remaining tumor tissue continued despite the chemotherapy. The chemotherapy regimen was altered to paclitaxel and carboplatin. Following one course, the remaining tumor increased further, causing obstruction to the bilateral ureters and leading to bilateral hydronephrosis. A double-J catheter was inserted into the right ureter and this was followed by supportive care. The patient is currently alive with the disease at 10 months following the surgery.

## Discussion

Adenoma malignum of the uterine cervix was initially described by Gusserow in 1870 (4) and, in 1963, McKelvey and Goodlin reported five cases of adenoma malignum (5). In 1975, the name MDA was proposed by Silverberg and Hurt (6) to more accurately reflect the resemblance of the glands to the normal endocervical glands and the lack of malignant cellular features. MDA is a rare subtype of mucinous adenocarcinoma of the uterine cervix, accounting for only 1–3% of uterine cervical adenocarcinoma and 0.15–0.45% of all cervical carcinomas of the uterus reported in the literature (1). The major clinical manifestations of MDA are profuse or mucoid discharge and irregular vaginal bleeding (7).

The diagnostic methods of MDA are cytological evaluations, biopsies, cross-sectional imaging and immunostaining. MDA appears in MRI as a multicystic mass, demonstrating extremely high-signal intensity on T2-weighted images and isointensity or moderate hyperintensity on T1-weighted images, which extends from the endocervical glands to the deep cervical stroma, with solid portions located deep in the endocervix (8,9). MDA is difficult to diagnose as few findings conclusively suggest malignancy on cytological or histological examination. MDA lesions are located deep in the endocervix and exhibit an endophytic growth pattern, which also makes it difficult to determine an accurate cytological and histological diagnosis (10). Li *et al* (7) reported that the detection rate of MDA by cytological evaluation was only 32.7%. Granter and Lee (11) reported that enlarged glandular cells in honeycombed sheets with abundant cytoplasm in exfoliative cytology were crucial in the detection of MDA; however, a preoperative histological diagnosis of MDA is also often difficult to determine. Preoperative punch biopsy occasionally fails to confirm the diagnosis of MDA as the tumor glands positioned in the deep cervical stroma require a deep biopsy (i.e. cervical conization) to confirm the presence of invasion. The monoclonal antibody HIK-1083, which detects mucin produced and secreted by the gastric mucous cells, has been indicated to be useful in the correct diagnosis of MDA (12). However, endocervical glandular hyperplasia with pyloric gland metaplasia has also been observed to be positive for HIK-1083 (13). Li *et al* (7) reported that the detection rate of MDA following a single biopsy was 28.7% and the rate of the total number of biopsies including cervical conization was 50.7%. Treatment and prognosis of MDA are controversial. Surgical treatment is the most successful option; however, no standard surgical treatment or adjuvant therapy have been established. The treatment of MDA frequently shadows that of endocervical adenocarcinoma and despite the presence of well-differentiated histopathological features, the prognosis of MDA is poor. This is due to the likelihood of the tumor to exhibit lymph node involvement and the early presence of peritoneal carcinomatosis, in contrast to the behavior of classical adenocarcinoma (5). However, a number of studies have reported a favorable prognosis (7,14) and stated that a poor prognosis of MDA is not always observed if it is identified at an early stage. The present case exhibited peritoneal carcinomatosis and distant metastasis to the left supraclavicular lymph node at diagnosis; therefore, optimal surgery could not be performed and the two types of adjuvant chemotherapy were ineffective.

McGowan *et al* (2) reported that Peutz-Jeghers syndrome may complicate MDA (2), and Gilks *et al* (15) reported that approximately half of all MDAs are complicated with ovarian mucinous tumors. The present case, however, was not complicated with these diseases.

The current study describes a rare case of MDA that was difficult to differentiate from endometrial adenocarcinoma of the corpus uteri preoperatively; following the diagnosis of endometrial cancer, surgery was performed. The tumor was diagnosed as MDA of the uterine cervix following pathological examination of the hysterectomy specimen. In cases where mild thickening of the endometrium is observed and the endometrial biopsy results indicate a well-differentiated adenocarcinoma, with cystic lesions in the uterine corpus and uterine cervix, the possibility of the invasion of MDA of the uterine cervix to the uterine corpus must be considered.
